# Living in Cold Blood: *Arcobacter, Campylobacter*, and *Helicobacter* in Reptiles

**DOI:** 10.3389/fmicb.2019.01086

**Published:** 2019-05-15

**Authors:** Maarten J. Gilbert, Birgitta Duim, Aldert L. Zomer, Jaap A. Wagenaar

**Affiliations:** ^1^Department of Infectious Diseases and Immunology, Faculty of Veterinary Medicine, Utrecht University, Utrecht, Netherlands; ^2^Reptile, Amphibian and Fish Conservation Netherlands, Nijmegen, Netherlands; ^3^WHO Collaborating Center for Campylobacter/OIE Reference Laboratory for Campylobacteriosis, Utrecht, Netherlands; ^4^Wageningen Bioveterinary Research, Lelystad, Netherlands

**Keywords:** *Arcobacter*, *Campylobacter*, *Helicobacter*, Epsilonproteobacteria, reptile, biodiversity, ecology, evolution

## Abstract

Species of the Epsilonproteobacteria genera *Arcobacter, Campylobacter*, and *Helicobacter* are commonly associated with vertebrate hosts and some are considered significant pathogens. Vertebrate-associated Epsilonproteobacteria are often considered to be largely confined to endothermic mammals and birds. Recent studies have shown that ectothermic reptiles display a distinct and largely unique Epsilonproteobacteria community, including taxa which can cause disease in humans. Several *Arcobacter* taxa are widespread amongst reptiles and often show a broad host range. Reptiles carry a large diversity of unique and novel *Helicobacter* taxa, which apparently evolved in an ectothermic host. Some species, such as *Campylobacter fetus*, display a distinct intraspecies host dichotomy, with genetically divergent lineages occurring either in mammals or reptiles. These taxa can provide valuable insights in host adaptation and co-evolution between symbiont and host. Here, we present an overview of the biodiversity, ecology, epidemiology, and evolution of reptile-associated Epsilonproteobacteria from a broader vertebrate host perspective.

## Introduction

With the advent of next generation sequencing techniques, recent years have seen growing research possibilities in bacterial biodiversity, ecology, epidemiology, and evolution. Especially the interplay between the microbiota and the vertebrate host has received much attention. Although vertebrate-associated Epsilonproteobacteria, primarily comprising taxa of the genera *Arcobacter, Campylobacter*, and *Helicobacter*, usually form a relatively small part of the vertebrate microbiota based on species abundance, they include some significant pathogens, such as *Campylobacter jejuni* and *Helicobacter pylori*. However, many of those species may be pathogenic in a particular host, while having a commensal or even mutualistic relationship with other hosts (Hermans et al., 2012; Lin and Koskella, 2015). Due to a varying degree of host association and adaptation, both generalists and specialists can be recognized.

Much focus has been on the microbiota of avian and mammalian hosts, while reptiles, which form a physiologically distinct, yet evolutionary intermediate group of amniotic vertebrates between mammals and birds, are relatively understudied. Although diverse and paraphyletic when omitting the related birds, reptiles are often grouped together based on shared features, such as a dependency on external heath sources (ectothermy). Compared to endothermic birds and mammals, ectothermic reptiles show a distinct physiology. Reptiles often show considerable fluctuations and a lower average body temperature compared to most endothermic vertebrates. Although ectothermic, reptiles are phylogenetically closely related to birds. As such, Epsilonproteobacteria species composition in reptiles could be either determined by physiology or phylogeny. Characteristics shared by nearly all reptiles, such as an ectothermic lifestyle, could select for a microbiota distinct from the microbiota encountered in endothermic vertebrates and might obscure phylo-genic ancestry. Due to the distinct phylogeny and physiology compared to birds and mammals, reptiles can provide valuable insights in Epsilonproteobacteria host adaptation and co-evolution.

Not much is known about the biodiversity, ecology, epidemiology, and evolution of reptile-associated Epsilonproteobacteria. These aspects will be explored in this review from a broader vertebrate host perspective.

## Epsilonproteobacteria Diversity and Taxonomy

**FIGURE 1:**
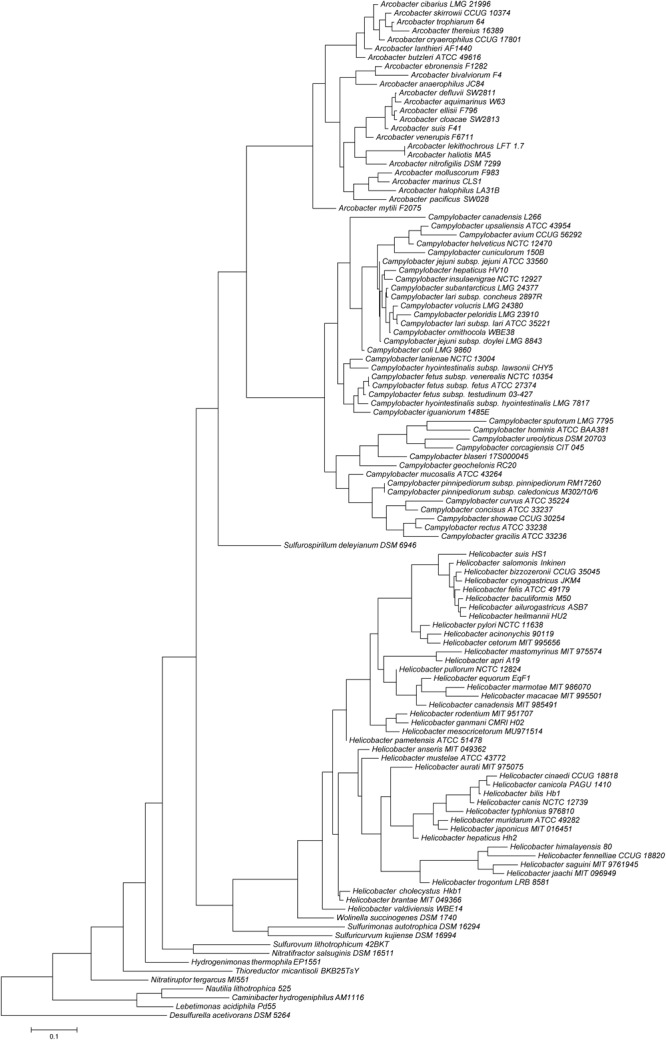
Epsilonproteobacteria 16S rRNA gene phylogeny, including all *Arcobacter, Campylobacter*, and *Helicobacter* species and the type species of the remaining genera. *Desulfurella acetivorans* was used as outgroup and root of the tree.

The epsilon subclass of the Proteobacteria (Epsilonproteobacteria) comprises high species diversity. Members of the Epsilonproteobacteria have been found in a wide range of niches, varying from deep-sea vents to plant roots and the vertebrate gastrointestinal tract. Most vertebrate-associated species belong to the genera *Arcobacter, Campylobacter, Helicobacter*, and *Wolinella* (Figure 1). In contrast to *Arcobacter*, of which several members are free-living in the environment or associated with non-vertebrate hosts, *Campylobacter* and *Helicobacter* are predominantly associated with vertebrate hosts. All these genera consist of Gram-negative, often spiral or curved rod-shaped and flagellated, motile bacteria. Most members grow optimally at reduced oxygen levels (microaerobic atmosphere), although some members grow anaerobically, and especially members of the *Arcobacter* genus can grow aerobically. All currently known vertebrate-associated species are primarily associated with the digestive tract or the reproductive tract. Most members are considered commensals, but pathogenicity in humans and other animals varies per species or strain. Important pathogens in humans include *Campylobacter jejuni*, which is a leading cause of gastroenteritis in many countries (Olson et al., 2008), and *Helicobacter pylori*, the bacterial agent associated with gastric ulcers and gastric cancer (Parsonnet et al., 1991). A noteworthy pathogen in animals is *Campylobacter fetus*, which can cause fertility problems and abortion, predominantly in ruminants, but can also cause occasional infection in humans, often with a systemic component in people with underlying illness (Blaser et al., 2008; Wagenaar et al., 2014). Besides these species, many other species have been associated with disease as well (Solnick and Schauer, 2001; Debruyne et al., 2008; Collado and Figueras, 2011).

The number of validly described *Campylobacter, Arcobacter*, and *Helicobacter* species has increased steadily. *Campylobacter fetus*, initially described as *Vibrio fetus*, was the first Epsilonproteobacteria species described (Smith and Taylor, 1919). With the discovery of novel species and introduction of sequence-based methods several highly divergent clades emerged, which demanded a thorough revision of *Campylobacter* taxonomy. *Campylobacter* was split in two families: the *Campylobacteraceae*, comprising the genera *Arcobacter, Campylobacter*, and *Sulfurospirillum*, and in the *Helicobacteraceae*, comprising the genera *Helicobacter* and *Wolinella* (Goodwin et al., 1989; Vandamme et al., 1991). Based on recent genomic analyses it was found that the Epsilonproteobacteria were not monophyletic with all other classes of Proteobacteria, and reassignment of this group to the novel phylum Campylobacterota has been proposed (Waite et al., 2017). Additionally, the genera *Arcobacter* and *Sulfurospirillum* were removed from the family *Campylobacteraceae* and reassigned to the novel families *Arcobacteraceae* and *Sulfurospirillaceae*, respectively, and *Helicobacter* and *Wolinella* are the only genera in the family *Helicobacteraceae*. In addition, the genera *Campylobacter* and *Helicobacter* consist of several distinct clades which may represent separate genera. Pending more research in support of these reassignments, the current taxonomy will be used here. In total, 24 *Arcobacter* species, 31 *Campylobacter* species, and 41 *Helicobacter* species have been validly described at the moment of writing^1^.

## Epsilonproteobacteria Ecology

In general, vertebrate-associated *Arcobacter* and, to a lesser extent, *Campylobacter* species show a broad host association, whereas most *Helicobacter* species show a more confined host association. These differential host dependencies are reflected in the genome sizes of these different genera, with a certain degree of genomic reduction observed in the vertebrate host-adapted *Campylobacter* and *Helicobacter*, which is less pronounced in *Arcobacter*. *Arcobacter* genomes encode a more diverse array of pathways than *Campylobacter* and *Helicobacter*, which often show incomplete pathways or lack certain pathways completely.

The ecology and host association of *Arcobacter, Campylobacter*, and *Helicobacter* can partly be explained by their physical properties. None of these genera are forming endospores and all are depending on moist conditions, making them susceptible to desiccation.

The genus *Arcobacter* comprises free-living species and animal- and plant-associated species. The habitat of *Arcobacter* members is highly diverse. Species have been isolated from sewage, estuarine sediments, marine and hyper saline environments, plant roots, and animals, including bivalve mollusks (Collado and Figueras, 2011). This habitat can be explained by certain characteristics shared by most species. Most members of the genus *Arcobacter* are able to grow at atmospheric oxygen levels. Many show an optimum growth at lower temperatures than *Campylobacter* and *Helicobacter* (18–37°C), and show no or less optimal growth at higher temperatures (37–42°C) (On et al., 1996). Relatively few *Arcobacter* species are associated with vertebrate hosts.

All *Campylobacter* species are associated with vertebrate hosts and many species show a broad host range. Members of this genus have been isolated from the vertebrate digestive tract and reproductive organs. Intestinal *Campylobacter* species show a strong affinity to the intestinal mucosa. As such, members of the *Campylobacter* genus show no or little growth at atmospheric oxygen levels; most prefer a microaerobic atmosphere and some grow at anaerobic conditions. This genus shows a wide growth temperature range, from 18 to 42°C, but most members show growth at 30–37°C (On et al., 1996). Due to its implication in human disease, most research has focused on *C*. *jejuni*.

All *Helicobacter* species are associated with vertebrate hosts and often show high host specificity. *Helicobacter* species are not or rarely isolated from environmental sources. Within the genus *Helicobacter* urease producing gastric species and enterohepatic species are recognized. *Helicobacter* species are commonly present as a part of the vertebrate gastric, enteric, and hepatobiliary biota (Solnick and Schauer, 2001). All members of the *Helicobacter* genus show no or little growth at atmospheric oxygen levels; most prefer a microaerobic or anaerobic atmosphere. Most species show optimal growth at 37–42°C (On et al., 1996). As a human pathogen, most research has focused on the gastric species *H*. *pylori*, which shows an intricate relationship with its human host (Atherton and Blaser, 2009).

## The Reptilian Host

Within the extant amniotic vertebrates, reptiles comprise a diverse class of animals, which are grouped together based on several internal and external characteristics shared by all or most members. In general, whereas mammals and birds are endothermic and have more constant body temperatures (homeothermic), reptiles are ectothermic and largely dependent on external heat sources for their preferred body temperatures, which can show considerable fluctuations (poikilothermic). Within the extant reptiles three major lineages can be discerned: Crocodilia (crocodiles); Lepidosauria, which consists of Sphenodontia (tuatara) and Squamata (lizards and snakes); and Testudines (chelonians). However, based on phylogeny, extant reptiles are considered paraphyletic as birds are excluded from reptilian phylogeny; the most recent common ancestor of all extant reptiles is the ancestor of all birds as well. In this respect, crocodilians are an interesting group to analyze the Epsilonproteobacteria species composition and co-evolution, since they are phylogenetically closer related to birds than to other ectothermic reptiles, but in contrast to endothermic birds display an ectothermic lifestyle. However, the Epsilonproteobacteria composition of crocodilians is poorly known.

Most of the extant reptiles are carnivorous, especially snakes and crocodiles, although the diet is supplemented by a certain amount of plant matter in many lizards and chelonians, and some species, especially tortoises, are considered herbivorous. As has been shown for other vertebrates (Ley et al., 2008), the diet and associated physiology of the gastrointestinal tract might be important factors in determining the intestinal microbiota composition.

Also, the host defense system might also play an important role in the microbiota composition. The reptilian immune system is distinct from the avian and mammalian immune system on several aspects. In contrast to birds and mammals, reptiles do not form germinal centers and lack Peyer’s patches, aggregations of lymphoid tissue which sample antigen directly from the lumen of the intestinal tract and are usually found in the lowest part of the small intestine (Zimmerman et al., 2010). Little is known about the adaptive immune response of reptiles. However, the innate immune system, including components such as non-specific leukocytes, antimicrobial peptides, the complement system, and toll-like receptors, responds quickly as a non-specific first line of defense against a broad range of pathogens and in many cases the responses are stronger than those of mammals (Zimmerman et al., 2010), although this may vary depending on the pathogen (Voogdt et al., 2016).

## The Reptilian Bacterial Microbiota

Relatively little is known about the reptilian intestinal microbiota. Amongst others, the intestinal microbiomes of the Galápagos land iguana (*Conolophus subcristatus*), Galápagos marine iguana (*Amblyrhynchus cristatus*), *Liolaemus* species, *Phymaturus williamsi, Anolis* species, the Burmese python (*Python bivittatus*), American alligator (*Alligator mississippiensis*), and the green turtle (*Chelonia mydas*) have been determined by sequence-based methods (Costello et al., 2010; Hong et al., 2011; Keenan et al., 2013; Ren et al., 2016; Kohl et al., 2017; Ahasan et al., 2018). Some of these studies showed that the microbiota changed drastically upon food intake after periods of fasting (Costello et al., 2010; Keenan et al., 2013). At the phylum level, Firmicutes and Bacteroidetes dominated the fecal microbiota of most species, but Fusobacteria dominated in American alligators, which is unique amongst mammals and reptiles, and which might reflect their archosaur ancestry (Keenan et al., 2013). However, in green turtles (*Chelonia mydas*) Proteobacteria were dominating and *Campylobacter fetus* was significantly abundant (Ahasan et al., 2018). A bacterial phylum-level analysis of fecal microbiome data showed that microbiome compositions did not reflect host phylogenetic affiliations or diet for reptiles (Keenan et al., 2013), although a significant correlation between diversity of the intestinal bacterial microbiota and phylogeny was observed in related *Anolis* species, suggesting that the radiation of this host lineage has influenced the diversification of their microbiota (Ren et al., 2016). In mammals it has been shown that host diet and phylogeny both influence bacterial diversity, which increases from carnivory to omnivory to herbivory, and that bacterial communities co-diversified with their hosts, which could be explained by effective vertical transmission in mammals (Ley et al., 2008; Ochman et al., 2010). As in mammals, a more diverse intestinal microbiota is expected as well in hindgut fermenting omnivorous and herbivorous reptiles.

## Reptiles as a Newly Detected Host for Epsilonproteobacteria

The Epsilonproteobacteria genera *Campylobacter, Arcobacter*, and *Helicobacter* are frequently isolated from various vertebrate hosts, primarily mammals and birds. The description of *C*. *fetus* from a chelonian during the eighties of the last century was the first Epsilonproteobacteria species reported from reptiles (Harvey and Greenwood, 1985). After that initial finding more *Arcobacter, Campylobacter*, and *Helicobacter* species have been reported to be isolated from reptiles. *Arcobacter butzleri* and a *Helicobacter* species have been isolated from tortoises (Debruyne et al., 2008; Stacy and Wellehan, 2010). A genetically distinct variant of *Campylobacter fetus* was isolated from reptiles and from humans that had direct or indirect contact with reptiles (Harvey and Greenwood, 1985; Tu et al., 2004; Dingle et al., 2010; Wang et al., 2013). This reptile-associated *C*. *fetus* has been shown to cause infection in humans with underlying disease (Tu et al., 2004). Another *Campylobacter fetus*-like species was isolated from the feces of a sick leopard tortoise (*Stigmochelys pardalis*) (Benejat et al., 2014). Following these case reports, it was shown that reptiles comprise a significant reservoir for *Arcobacter, Campylobacter, Helicobacter*, and *Sulfurospirillum* species, including potential novel species (Gilbert et al., 2014). The predominating *Campylobacter* taxa in reptiles, *C*. *fetus* subsp. *testudinum* and *C*. *iguaniorum*, have been described as a novel subspecies and species, respectively (Fitzgerald et al., 2014; Gilbert et al., 2015). Another recently described species, *C*. *geochelonis*, has also been isolated exclusively from reptiles (Gilbert et al., 2014). Despite the high prevalence, *C. iguaniorum* has never been reported before, whereas the required conditions for isolation are similar to the methods used for species such as *C*. *jejuni*. This suggests that reptiles have been overlooked as potential host for Epsilonproteobacteria.

## Species Diversity of Reptile-Associated Epsilonproteobacteria

In total, three *Arcobacter* species (*A*. *butzleri, A*. *cryaerophilus*, and *A*. *skirrowii*), four *Campylobacter* species (*C*. *fetus, C*. *geochelonis, C*. *hyointestinalis*, and *C*. *iguaniorum*), ten putative novel *Helicobacter* species, and one putative novel *Sulfurospirillum* species have been isolated from reptiles (Lawson and Owen, 2007; Stacy and Wellehan, 2010; Gilbert et al., 2014, 2017; Piccirillo et al., 2016). *Sulfurospirillum* was isolated once from a chelonian and, apart from this isolate, the *Sulfurospirillum* genus solely consists of members which have been isolated from environmental sources, making it likely that this *Sulfurospirillum* represents an incidental introduction into a vertebrate host.

The known Epsilonproteobacteria diversity is largely based on studies in humans and production animals, which is only a small fraction of potential hosts. Over the last years more Epsilonproteobacteria species have been described from vertebrate hosts other than humans or companion animals, suggesting a large potential reservoir of undiscovered Epsilonproteobacteria species. This also indicates that the number of validly described Epsilonproteobacteria species is largely limited by the research efforts outside known reservoirs. This is also hampered by the fastidious nature of many vertebrate-associated Epsilonproteobacteria, as the deposit of cultured bacteria in culture collections is mandatory for valid description of a novel species. However, with increasing interest in screening of novel potential reservoirs and especially improved sequencing methods, enabling metagenomics approaches, a large increase in the known Epsilonproteobacteria diversity is expected. With an increase in known diversity, the phylogenetic resolution increases and novel clades might appear.

## Thermotolerance

Both *Campylobacter* and *Helicobacter* include members adapted to either high or low temperatures within the optimum temperature range observed in most birds, mammals, and reptiles. As *Campylobacter* and *Helicobacter* form distinct clades, low temperature adaptation has likely evolved independently in both genera. Especially in *Helicobacter*, this seems to be related to a high level of adaptation to a poikilothermic reptilian host (Gilbert et al., 2017). Low temperature adaptation is prevailing in many other members of the *Campylobacteraceae* (e.g., free-living *Arcobacter* and *Sulfurospirillum*) and high temperature adaptation might be exceptional. Adaptation to extreme environments is well conserved amongst Epsilonproteobacteria, which have been shown associated with deep-sea thermal vents and hypersaline environments (Donachie et al., 2005; Nakagawa et al., 2005).

No thermotolerant *Campylobacter* species have been isolated from reptiles (Wang et al., 2013; Gilbert et al., 2014). The *Campylobacter* species isolated from reptile are all low temperature adapted species from the same clade (*C*. *fetus* group). In contrast to other *Campylobacter* taxa, all share high homology in their NADH:quinone oxidoreductase complex I subunits (NuoA-N) with low temperature adapted *Arcobacter* (Gilbert et al., 2016a). Indeed, in other organisms NADH:quinone oxidoreductase complex I is considered the most thermolabile protein complex of oxidative phosphorylation (Downs and Heckathorn, 1998). It is tempting to speculate that the high homology observed in the NADH:quinone oxidoreductase complex I subunits reflects parallel evolution which might be related to low temperature adaptation. Amphibians could provide significant insights into bacterial thermal adaptation in poikilothermic hosts. Being non-amniotic vertebrate tetrapods, amphibians are only distantly related to reptiles, although they are often grouped together due to superficial external resemblance and a shared poikilothermic lifestyle. No Epsilonproteobacteria have been reported to be isolated from amphibians. Own results based on culturing and PCR indicate that no or few Epsilonproteobacteria are present in amphibians (unpublished data). Parallels exist with *Salmonella*, a generalist species which is frequently isolated from mammals, birds and reptiles, but not or rarely from amphibians (Briones et al., 2004). This indicates that a poikilothermic nature is not the only predictor in the composition of the poikilothermic vertebrate microbiota and precludes a general thermal adaptation irrespective of host group phylogeny. As for many reptiles, the amphibian diet consists mainly of invertebrates, and although the amphibian intestinal microbiome resembles the amniote microbiome (Kohl et al., 2013), it appears to be less diverse and lacking certain bacterial species typical for reptiles. This suggests that host physiology is important in the microbiome composition and that at least some level of host adaptation occurs, even in generalist species such as *Salmonella*.

## Epsilonproteobacteria Host Association

The presence of *Arcobacter* and *Campylobacter* in a large diversity of reptiles (some taxa have been isolated from lizards, snakes, and chelonians) is indicative of a generalist lifestyle, rather than a specialist lifestyle. Epsilonproteobacteria with a high survival rate outside the vertebrate host, such as some *Arcobacter* species, tend to show a more relaxed host association than Epsilonproteobacteria with a higher level of host specificity, such as many *Helicobacter* species. However, by adaptation to a specific niche, the latter can exploit unique resources and might show a competitive advantage and higher survival in its specific niche than generalist species, as can be seen in gastric *Helicobacter* species (Table 1).

**Table 1:** Overview of the Epsilonproteobacteria taxa isolated from reptiles and respective host association.

	Reptiles			Mammals	Birds	References
	Lizards	Snakes	Chelonians			
**Arcobacter**						
*Arcobacter butzleri*	+	+	+	+	+	Gilbert et al., 2014
*Arcobacter cryaerophilus*	+	–	+	+	+	Gilbert et al., 2014
*Arcobacter skirrowii*	–	–	+	+	+	Gilbert et al., 2014
**Campylobacter**						
*Campylobacter fetus* subsp. *testudinum*	+	+	+	–^∗∗∗^	–	Harvey and Greenwood, 1985; Wang et al., 2013; Gilbert et al., 2014
*Campylobacter hyointestinalis*	–	–	+	+	–	Gilbert et al., 2014
*Campylobacter iguaniorum*	+	–	+	–	–	Benejat et al., 2014; Gilbert et al., 2014
*Campylobacter geochelonis*	–	–	+	–	–	Gilbert et al., 2014; Piccirillo et al., 2016
**Helicobacter**						
*Helicobacter* 11S02596-1^∗^	+	–	–	–	–	Gilbert et al., 2014, 2017
*Helicobacter* 11S02629-2^∗^	–	–	+	–	–	Gilbert et al., 2014, 2017
*Helicobacter* 11S03491-1^∗^	+	–	–	–	–	Gilbert et al., 2014, 2017
*Helicobacter* 12S02232-10/SP2^∗^	+	+	–	–	–	Lawson and Owen, 2007; Gilbert et al., 2014, 2017
*Helicobacter* 12S02256-12^∗^	+	–	–	–	–	Gilbert et al., 2014, 2017
*Helicobacter* 12S02634-8^∗^	+	–	–	–	–	Gilbert et al., 2014, 2017
*Helicobacter* 13S00401-1^∗^	–	–	+	–	–	Gilbert et al., 2014, 2017
*Helicobacter* 13S00482-2^∗^	+	–	–	–	–	Gilbert et al., 2014, 2017
*Helicobacter* 13S00477-4^∗^	+	–	–	–	–	Gilbert et al., 2014, 2017
*Helicobacter* ‘pancake tortoise’^∗^	–	–	+	–	–	Stacy and Wellehan, 2010
**Sulfurospirillum**						
*Sulfurospirillum* 11S05485-2^∗^	–	–	+^∗∗^	–	–	Gilbert et al., 2014

*^∗^Putative species based on 16S rRNA. ^∗∗^Only known *Sulfurospirillum* taxon isolated from a vertebrate. ^∗∗∗^Occasionally isolated from humans which had been exposed to reptiles in most cases.*

Some *Campylobacter* species can be found in many different host species, such as *C. jejuni* and *C. coli*. Other species are more associated, or even restricted, to certain hosts such as *C. upsaliensis* in dogs, *C. helveticus* in cats, *C. insulaenigrae* and *C*. *blaseri* in seals, and *C. fetus* subspecies *fetus* and *venerealis* in ruminants. *C*. *fetus* subsp. *testudinum* and *C*. *iguaniorum* are predominantly isolated from poikilothermic reptiles. At least part of the captive-held reptiles that were screened for Epsilonproteobacteria were living near birds in zoos or were fed on poultry and were most likely exposed to thermotolerant *Campylobacter* species, such as *C*. *coli* and *C*. *jejuni*. Nevertheless, despite a generalist lifestyle and broad host range in mammals and birds, combined with suitable culturing methods, these thermotolerant *Campylobacter* species have not been isolated from reptiles (Gilbert et al., 2014). This shows that, at least for *Campylobacter*, a distinct host dichotomy is observed at species level. Indeed, comparisons of growth temperature ranges confirmed that most *Campylobacter* species associated with reptiles can grow at lower temperatures than *Campylobacter* species associated with mammals and birds (Gilbert et al., 2015). It could be speculated that this is an adaptation to the body temperature encountered in these different hosts. Although the underlying factors for this distinct host association are not entirely clear, genomic studies provide clues about the genetic background of this differential host adaptation, such as the differential homology observed in the NADH:quinone oxidoreductase complex I subunits (Gilbert et al., 2016a,b).

## *Campylobacter fetus* Host Association

All highly prevalent *Campylobacter* species isolated from reptiles (*C*. *fetus, C*. *hyointestinalis*, and *C*. *iguaniorum*) are closely related and belong to the same phylogenetic clade. Of this clade, only *C*. *lanienae* has not been found in reptiles. Based on the currently known species distribution it is tempting to speculate about a potential reptilian origin of this particular *Campylobacter* clade. Especially the host preference of *C*. *fetus* is remarkable. A distinct host dichotomy is encountered within the species: while the subspecies *C*. *fetus* subsp. *fetus* is predominantly isolated from mammals, mostly ruminants, and *C*. *fetus* subsp. *venerealis* is restricted to the reproduction organs of cattle, *C*. *fetus* subsp. *testudinum* is predominantly isolated from reptiles (Table 1). Although, like *C*. *fetus* subsp. *testudinum*, both *C*. *fetus* subspecies *fetus* and *venerealis* can grow at low temperatures (20–25°C), these subspecies have never been isolated from reptiles. One of the factors shaping this host dichotomy, as shown by genomic analyses, may be the presence of a tricarballylate catabolism locus in *C*. *fetus* subsp. *testudinum*, which was absent from both mammal-associated *C*. *fetus* subspecies (Gilbert et al., 2016b). Interestingly, the tricarballylate catabolism locus was also present in reptile-associated *C*. *iguaniorum* and *Helicobacter* lineages (Gilbert et al., 2016a, 2017). This locus potentially enables *C*. *fetus* subsp. *testudinum* to use the citrate analog tricarballylate as carbon and energy source (Lewis et al., 2004). Tricarballylate is toxic to ruminants by inhibiting aconitase and the citric acid cycle (Russell, 1985). However, reptiles have been shown less susceptible to aconitase inhibition than mammals (McIlroy, 1986). As such, reptiles are expected to be more tolerant to tricarballylate, which might be more abundant in the reptilian intestine than in the mammalian intestine. The uptake of genetic material through lateral gene transfer can give a competitive advantage and the possibility to exploit new niches (Papke and Ward, 2004). In this way, the tricarballylate catabolism locus might have influenced the host adaptation and genetic divergence in *C*. *fetus* subsp. *testudinum*. However, a loss of this locus in mammal-associated *C*. *fetus* due to redundancy cannot be excluded. Furthermore, the homology between reptile- and mammal associated *C*. *fetus* based on average amino acid identity is roughly 95%, meaning that also other factors influencing host adaptation and genetic divergence in *C*. *fetus* might exist and reside in the 5% average amino acid difference.

Genetic diversity has been shown to be low for *C*. *fetus* subspecies *fetus* and *venerealis*, and a recent entry from another reservoir into the ruminant host reservoir followed by rapid expansion has been suggested (van Bergen et al., 2005; van der Graaf-van Bloois et al., 2016). Compared to *C*. *fetus* subspecies *fetus* and *venerealis, C*. *fetus* subsp. *testudinum* shows higher genetic diversity (Dingle et al., 2010; Gilbert et al., 2016b), suggesting a more ancient entry of *C*. *fetus* subsp. *testudinum* into the reptilian host reservoir and subsequent genetic divergence.

## Host Association of Reptile-Associated *Helicobacter*

Generally, there appears to be an increasing vertebrate host association within the genera *Arcobacter, Campylobacter*, and *Helicobacter*, respectively, although some *Helicobacter* species can occur in multiple hosts as well (Solnick and Schauer, 2001) (Table 1). This host association is nicely illustrated by *Helicobacter pylori*, which shows an intricate symbiosis with humans, the primary host for this species (Falush et al., 2003). In addition to this, compared to other vertebrate-associated Epsilonproteobacteria genera, the higher number of distinct *Helicobacter* taxa isolated from many different vertebrate hosts species suggests a higher level of host adaptation and specificity. Also in reptiles the *Helicobacter* diversity (nine putative species) was higher than *Arcobacter* and *Campylobacter* diversity (three species of both genera) (Gilbert et al., 2014, 2017).

In contrast to *Arcobacter* and *Campylobacter*, of which most taxa were isolated from all reptilian orders examined, *Helicobacter* showed stricter host association. Based on 16S rRNA and whole genome phylogeny a *Helicobacter* clade associated with lizards and a clade associated with chelonians was observed (Gilbert et al., 2014, 2017). These clades were distinct from mammalian and avian *Helicobacter* species, indicating long-term divergence in a reptilian host. The lizard-associated *Helicobacter* lineages carried a urease locus and were most closely related to gastric *Helicobacter* species, while the chelonian-associated *Helicobacter* species were most closely related to enterohepatic *Helicobacter* species. This indicated initial adaptation to an anatomical niche and subsequent diversification in either a lizard or chelonian host type. The phylogeny of reptile-associated *Helicobacter* paralleled association with either a lizard or chelonian host, indicating a high level of host specificity. The high diversity and deep branching within these clades supported long-term coevolution with, and extensive radiation within the respective reptilian host type.

The reptile-associated *Helicobacter* lineages were able to grow at lower temperatures (25°C) and a larger temperature range (25–42°C) than *Helicobacter* species from birds and mammals, which might reflect thermal adaptation to a reptilian host (Lawson and Owen, 2007; Gilbert et al., 2017). As growth at lower temperatures was observed in both the distantly related chelonian- and in the lizard-associated *Helicobacter* clade, and mammal-associated *Helicobacter* clades were more basal to these, the ability to grow at lower temperatures likely originated at least twice in *Helicobacter* evolution.

As in reptile-associated *Campylobacter* taxa, a tricarballylate catabolism locus was present in all *Helicobacter* lineages from reptiles, while it was rarely present in *Helicobacter* species isolated from other host types, indicating that this locus might confer a competitive advantage in a reptilian host. Noteworthy, one of the genes of this locus, *tcuC*, showed higher homology between *Campylobacter fetus* subsp. *testudinum, C*. *iguaniorum* and the chelonian-associated *Helicobacter* lineages than the latter and the lizard-associated *Helicobacter* lineages. This suggests that recombination of the tricarballylate catabolism locus has occurred between *Helicobacter* and *Campylobacter*, potentially in a chelonian host.

## Transmission and Survival

Efficient transmission between hosts is crucial for long-term survival of host-adapted bacterial species. Most vertebrate-associated Epsilonproteobacteria rely on a host for long-term survival and show limited viability in the hostile environment outside the host. Indeed, while *Campylobacter* species can survive in the environment, they are generally not considered to propagate outside of an animal host (Rollins and Colwell, 1986). Some *Campylobacter* species can persist in moist and aquatic environments, and *Campylobacter* species can be transmitted via feces contaminated water sources (Baily et al., 2014). Also for *Helicobacter* species an environmental reservoir is often not known (Solnick and Schauer, 2001). In contrast, vertebrate-associated *Arcobacter* species can survive in diverse environments and seem to be less dependent on a host for long-term survival (Miller et al., 2007; Ferreira et al., 2016). At least for *Campylobacter* and *Helicobacter*, the vertebrate host itself is likely the most important vector.

Transmission can occur from parent to offspring (vertical transmission) or from other sources (horizontal transmission). From a host perspective, transmission is dependent on physiology and contact structures of the host. In mammals, which are viviparous and display intensive parental care, contact structures between parent and offspring are more frequent, enabling efficient vertical transmission of the intestinal microbiota. Although oviparous, most birds show parental care, facilitating vertical transmission of bacteria. In reptiles, which are mostly oviparous and display no or little parental care, vertical transmission is likely less efficient than in mammals and birds. These physiological and behavioral disparities can potentially influence the composition and transmission of the reptilian intestinal microbiota, including the Epsilonproteobacteria. However, several physiological homologies can be found in reptiles and birds, such as oviparous reproduction and having a cloaca, a common distal part of the intestinal, reproductive, and urinary tracts.

The transmission of the intestinal microbiota in reptiles is poorly studied, but relatively well studied for avian intestinal bacterial genera which also occur in reptiles, such as *Campylobacter* and *Salmonella*, and parallels to reptiles might exist. Although *Campylobacter* species are found in the reproductive tracts of poultry, no or little *C*. *jejuni* is found in eggs and hatchlings (Camarda et al., 2000; Buhr et al., 2002; Cole et al., 2004). Direct vertical transmission of *Campylobacter* is considered absent or rare in birds and offspring will become colonized by *Campylobacter* after birth by fecal-oral transmission (Shanker et al., 1986; Sahin et al., 2002; Callicott et al., 2006). In contrast to *Campylobacter*, vertical transmission of *Salmonella* is possible, both by penetrating the egg in the ovaria and by cloacal contamination of the surface during oviposition, and has been shown in poultry (Gantois et al., 2009). A similar mode of infection in reptiles seems plausible. Indeed, penetration of turtle eggs by *Salmonella* Braenderup has been shown (Feeley and Treger, 1969). Most reptilian eggs are more fibrous and less calciferous compared to the avian egg and are usually highly permeable to water. In most species water uptake by the egg after deposition is crucial in development of the reptilian embryo. For this reason reptile eggs are usually deposited in moist locations. Both the permeable eggshell as the moist incubation could be favorable for vertical transmission of the intestinal microbiota, including the small and often highly motile Epsilonproteobacteria. Furthermore, many lizards and snakes are viviparous or ovoviviparous, giving birth to completely developed young. These factors could promote vertical transmission. Indeed a significant proportion of the intestinal microbiome overlapped between mother and recently born offspring in a live bearing lizard species, suggesting vertical transmission, although environmental transmission could not be excluded (Kohl et al., 2017). As in birds, horizontal transmission is likely important, at least in captivity. Indeed, molecular genotyping indicated that captive, unrelated reptile species having direct or indirect contact can harbor the same specific Epsilonproteobacteria variants (Gilbert et al., 2014). This phenomenon was observed in animals kept in the same housing, animals from the same zoo, and animals originating from the same shipment, indicating that horizontal transmission happens under these conditions. The exact transmission routes remain to be elucidated, but a fecal-oral transmission is likely, especially in captive animals and species displaying coprophagy. Many herbivorous chelonians display coprophagy, presumably to supplement their microbiota needed for hindgut fermentation. Also sexual transmission might play a role. In the common lizard (*Zootoca vivipara*) it was found that polyandrous females mating with multiple males harbored more diverse cloacal bacterial communities and differed more in community composition than did monandrous females, suggesting that the higher bacterial diversity found in polyandrous females is due to the sexual transmission of bacteria by multiple mates (White et al., 2011).

Regarding spatial transmission, migratory birds are an effective vector for avian-associated Epsilonproteobacteria species. Compared to birds, mammals and especially reptiles show less extensive migration patterns, which are likely influencing the distribution patterns of Epsilonproteobacteria associated with these hosts. This is nicely illustrated in *C. jejuni*, which occurs in many wild avian hosts and shows a pandemic distribution (Griekspoor et al., 2013).

To study these factors with little interference, free-living vertebrate populations with as little anthropogenic influences as possible might be favored over captive-held vertebrate populations. Captivity can influence the contact structures, transmission, and observed host association of vertebrate-associated Epsilonproteobacteria. Indeed, Epsilonproteobacteria prevalence has been shown higher in captive-held reptiles, although the number of sampled free-living reptiles was low (Gilbert et al., 2014). Nevertheless, Epsilonproteobacteria have also been identified in recently caught free-living reptiles, indicating the presence of Epsilonproteobacteria in free-living reptiles as well (Gilbert et al., 2014; Kohl et al., 2017; Ahasan et al., 2018). This is in concordance with studies examining *Salmonella* prevalence in reptiles, which showed a higher prevalence in captive-held reptiles than in free-living reptiles (Richards et al., 2004; Scheelings et al., 2011) and the introduction of *Salmonella* in captivity (Kohl et al., 2017). Many sampled captive-held reptiles were living in close contact with other animals of the same or other species in a density that is higher than under natural conditions. This may very well lead to other transmission dynamics and an intestinal microbiota composition distinct from free-living animals, as has been shown for mammals (Ochman et al., 2010). Interspecies transmission in unnatural reptile assemblies have been noted before for *Campylobacter* and *Helicobacter* (Schrenzel et al., 2010; Gilbert et al., 2014) and indicates that original host association, but also phylogenetic patterns of co-evolution between a vertebrate host and symbiont might be disturbed by anthropogenic influences.

## Virulence of Epsilonproteobacteria in Reptiles

The presence of Epsilonproteobacteria in many different reptiles without clinical symptoms indicates that most Epsilonproteobacteria taxa are carried without adverse health effects. Herbivorous reptiles (especially tortoises), which rely on their intestinal microbiota for hindgut fermentation and digestion of their vegetarian diet, showed highest Epsilonproteobacteria prevalence and diversity, without apparent adverse health effects. Little is known from literature about Epsilonproteobacteria and disease in reptiles. A *Helicobacter* species has been reported associated with septicemia in a pancake tortoise (*Malacochersus tornieri*), in which disseminated infection resulted in regional cellulitis and edema of the head and neck, and pericarditis (Stacy and Wellehan, 2010). However, closely related *Helicobacter* strains were isolated from tortoises without clinical symptoms, indicating that *Helicobacter* can be carried without adverse health effects (Gilbert et al., 2014). Furthermore, a *Helicobacter* species was found associated with septicaemia and mortality in wild blue iguanas (*Cyclura lewisi*) on Grand Cayman (Popescu, 2018).

*C*. *iguaniorum* has been isolated from the feces of a sick leopard tortoise (*Stigmochelys pardalis*), although this species was isolated from reptiles with and without clinical symptoms (Benejat et al., 2014; Gilbert et al., 2014, 2015). *C*. *fetus* subsp. *testudinum* was the predominantly isolated Epsilonproteobacteria species in snakes and, when present, was isolated in high numbers compared to presence in other reptiles (Gilbert et al., 2014). Noteworthy, all snakes carrying *C*. *fetus* subsp. *testudinum* suffered from often lethal intestinal infections. In humans, *C*. *fetus* is known for causing invasive infections with a systemic component (Blaser et al., 2008; Wagenaar et al., 2014), especially in elderly and immunocompromised (Tu et al., 2004; Patrick et al., 2013). If *C*. *fetus* subsp. *testudinum* is associated with infections in snakes, it may very well be that the animals were already weakened due to suboptimal living conditions in captivity, compared to the conditions in the wild, which may lead to malnutrition, stress, high parasite loads, and other disorders, leaving the animals more vulnerable to infection (Girling and Raiti, 2004). Indeed, very few bacteria have been implicated in reptile diseases as primary causative agents (Pasmans et al., 2008), which may be related to a generally more potent innate immune response in reptiles, compared to mammals (Zimmerman et al., 2010).

## Zoonotic Aspects of Reptile-Associated Epsilonproteobacteria

Most of the *Arcobacter* and *Campylobacter* species found in reptiles are associated with disease in humans, although infections are often sporadic and mainly affecting immunocompromised hosts (Lastovica and Allos, 2008; Collado and Figueras, 2011). Currently *C*. *fetus* is the only species for which an association between reptile contact and human infection has been demonstrated (Harvey and Greenwood, 1985; Tu et al., 2004; Patrick et al., 2013). Besides from humans, *C*. *fetus* subsp. *testudinum* has only been isolated from reptiles, with a reported culturing-based prevalence of 5.5–6.7% in reptiles and 7.1–9.7% in chelonians (Wang et al., 2013; Gilbert et al., 2014). Reptile-associated *C*. *fetus* subsp. *testudinum* showed a remarkable epidemiology, as all human cases were in men, most of whom were of Asian origin (Patrick et al., 2013). All patients with invasive *C. fetus* subsp. *testudinum* infections were >60 years of age or were immunocompromised. Humans may contract *C*. *fetus* subsp. *testudinum* though exposure to reptiles, possibly by ingestion or by contact with feces or the environment. Chelonians are frequently sold in Asian markets for consumption, which could explain the predominance of an Asian origin among reported patients. A GenBank search based on 16S rRNA revealed more human infections with *C*. *fetus* subsp. *testudinum* in China. Furthermore, in the period 2012–2013, at least 13 human cases were reported from the Guangzhou area alone (Hou et al., 2015). No epidemiological data were available for these cases, but since chelonians form an integral part of the Chinese cuisine, a reptilian origin is likely for these *C*. *fetus* subsp. *testudinum* infections. Based on multilocus sequence typing (MLST), *C*. *fetus* subsp. *testudinum* strains from human cases in China showed higher genetic diversity compared to those from the United States, and one strain isolated from a human (Hou et al., 2018) showed an identical sequence type (ST17) as a strain isolated from a chelonian (Dingle et al., 2010; Gilbert et al., 2016b), further affirming a reptilian origin for *C*. *fetus* subsp. *testudinum* infections in humans.

MLST and genomic data showed that, although geographically separated, all human invasive infections by *C*. *fetus* subsp. *testudinum* in the United States were caused by highly related strains (Dingle et al., 2010; Gilbert et al., 2016b). All invasive *C*. *fetus* subsp. *testudinum* strains uniquely shared a genomic region which was highly homologous in mammal-associated *C*. *fetus* and showed signs of recombination (Gilbert et al., 2016b). This region, *iamABC* (also annotated as *mlaFED*) is associated with invasion in *C*. *jejuni* and might be associated with invasion in these particular *C*. *fetus* subsp. *testudinum* strains as well (Carvalho et al., 2001). Furthermore, the systemic component in *C*. *fetus* infection has been shown associated with the surface array layer (S-layer), which protects the bacteria against a complement-based immune response and phagocytosis (Blaser et al., 2008). The S-layer encoding *sap* locus is highly variable, but can be divided in *sapA, sapB*, and occasional *sapAB*. Both in reptile- as in mammal-associated *C*. *fetus sapA* type strains are overrepresented in cases of septicemia, which might be related to the serum resistance of *sapA* type strains, as has been shown for mammal-associated *C*. *fetus* (Kienesberger et al., 2014). While the genetic diversity of *C*. *fetus* subsp. *testudinum* is larger in reptiles (Dingle et al., 2010; Wang et al., 2013; Gilbert et al., 2016b), only certain strains are associated with infection in humans in the United States. Besides genetic factors such as *iamABC*, this could also be related to differences in exposure to particular *C*. *fetus* subsp. *testudinum* strains. Nevertheless, the predominant occurrence of *C*. *fetus* infections in elderly and immunocompromised hosts stresses the importance of the immune system in preventing invasive infection (Blaser et al., 2008).

Of the other Epsilonproteobacteria species isolated from reptiles, *A*. *butzleri, A*. *cryaerophilus, A*. *skirrowii*, and *C*. *hyointestinalis* have all been associated with infections in humans. *C*. *iguaniorum* and the *Helicobacter* taxa have only been isolated from reptiles and have not been associated with infection in humans thus far, except for a case of bacteremia caused by a urease-negative *Helicobacter* strain, isolated from blood cultures of a 28-year-old man with X-linked agammaglobulinemia (Schwarze-Zander et al., 2010). Based on 16S rRNA homology, this *Helicobacter* strain was closely related to the *Helicobacter* clade isolated from chelonians (Stacy and Wellehan, 2010; Gilbert et al., 2014). Noteworthy, the patient had contact with poultry and with snakes, which were fed rodents, and, as this *Helicobacter* strain is related to the *Helicobacter* clade isolated from reptiles, the bacteremia caused by this *Helicobacter* strain might be related to exposure to snakes.

## Evolution of Vertebrate-Associated Epsilonproteobacteria

The current observed distribution of species is merely a cross section of an evolutionary process which has been going on since the beginning of life, and which is often progressing at speeds undetectable within a human lifespan. As such, each species can be in a different stage of speciation. As pioneered by Woese (1987) based on 16S rRNA, the prokaryotic divisions are ancient, as the evolutionary split of most divisions is pre-dating the origin of animals and often pre-dating the origin of eukaryotes. Also, bacteria hardly fossilize, so in order to reconstruct the bacterial evolutionary trajectory one has to rely on theoretical models based on genetic information. Nevertheless, the evolutionary trajectory for some highly host restricted (endosymbiont) bacterial species can be deduced from the evolutionary trajectory of its host species for which fossils are available, such as for *Buchnera* and their aphid host (Moran et al., 2008). Co-evolution between a host and its symbiont can be most apparent in case of congruent evolution. In that case both host and symbiont have similar phylogenetic trees. This has been reported for endosymbionts which are highly dependent on one host, are transmitted vertically, and do not have a free-living stage (Papke and Ward, 2004).

Most vertebrate-associated Epsilonproteobacteria are highly dependent on a vertebrate host for survival, and although no endosymbionts, they might show a phylogeny similar to their respective hosts depending on the level of host association. In general, vertebrate host association is highest in *Helicobacter*, followed by *Campylobacter* and *Arcobacter*, although differences between and within species can be observed. This is likely related to a decrease in survival outside the host, e.g., vertebrate-associated *Arcobacter* species more tolerant to atmospheric oxygen levels than *Campylobacter* and especially *Helicobacter*. This in turn might lead to isolation in a particular host and increased speciation, although this is also highly dependent on the host-associated transmission dynamics. This is reflected in the observed Epsilonproteobacteria diversity on species level in both reptiles and vertebrates in total, which is highest for *Helicobacter*, followed by *Campylobacter* and *Arcobacter*.

Indeed, for some vertebrate-associated Epsilonproteobacteria species a congruent evolution has been observed, such as for *H*. *pylori* and its human host (Falush et al., 2003; Linz et al., 2007). Considering the rather generalist lifestyle and broad host range of many *Campylobacter* species, highly similar phylogenies between *Campylobacter* and their vertebrate hosts are not to be expected. Nevertheless, *Campylobacter* is vertebrate host-adapted and although able to survive, they are considered unable to propagate outside this host (Rollins and Colwell, 1986). When examining the 16S rRNA-based phylogeny of the *Campylobacter* genus, at least three distinct clades consisting of more than one species can be discerned; *C*. *jejuni* and related species, *C*. *fetus* and related species, and *C*. *concisus* and related species (Figure 1). Interestingly, the *C*. *jejuni* clade mainly consists of thermotolerant bird-associated *Campylobacter* species, the *C*. *fetus* clade consists of non-thermotolerant reptile- and mammal-associated *Campylobacter* species, and the *C*. *concisus* clade solely consists of mammal-associated *Campylobacter* species. These clades are separated by deep branching in the phylogenetic tree and may even be considered distinct genera (Waite et al., 2017). Although host overlap between birds, mammals, and reptiles is frequent for these three *Campylobacter* clades, overall *Campylobacter* phylogeny is similar to the overall phylogeny of these vertebrate groups. One could speculate that these different *Campylobacter* clades are associated and co-evolving with these vertebrate groups since the last common ancestor of these amniotic vertebrate hosts diverged. However, there are also indications that the large-scale evolutionary pattern of *Campylobacter* and *Helicobacter* can be explained by initial adaptation to a particular (anatomical) niche and subsequent divergence in different vertebrate host types. For example, the *C*. *concisus* clade predominantly consists of oral *Campylobacter* species, while reptile-associated *Helicobacter* lineages do not form one separate clade distinct from mammal and bird-associated *Helicobacter* species, but instead form two separate clades, which are most closely related to, but distinct from, either enterohepatic or gastric *Helicobacter* species associated with mammals and birds (Gilbert et al., 2014, 2017).

For the vertebrate-associated Epsilonproteobacteria most evolutionary studies have focused on the human pathogens *H*. *pylori* and *C*. *jejuni*. Due to its high host specificity and efficient vertical transmission over a prolonged time, *H*. *pylori* and humans co-evolved and *H*. *pylori* phylogeny reflects human phylogeny (Falush et al., 2003; Linz et al., 2007). At least in some cases, gastric disease can be explained by disruption of the co-evolved human and *H*. *pylori* lineages (Kodaman et al., 2014). Although considered a human pathogen, *H*. *pylori* can be beneficial to its human host as well (Atherton and Blaser, 2009), illustrating an intricate long-term symbiosis. Nevertheless, no *Helicobacter* species closely related to human *H*. *pylori* were isolated from chimpanzees, our closest extant relatives, suggesting that the association between *H*. *pylori* and humans is of more recent times than the divergence time between both species (Moodley et al., 2012). This could be explained by host jumps between distantly related vertebrates, as *H*. *pylori* was most closely related to *H*. *acinonychis* from large felines (Eppinger et al., 2006). In contrast, *C*. *jejuni* shows a broad vertebrate host range. Birds are considered to be the primary host, which carry the bacteria without obvious clinical symptoms. *C*. *jejuni* has efficiently entered the agricultural niche and colonizes both avian and mammalian livestock. Mostly from this reservoir *C*. *jejuni* can colonize humans and cause infection. *C*. *jejuni* and the closely related *C*. *coli* both occur in the same agricultural niche and extensive genetic recombination and introgression between these two separate species has been shown (Sheppard et al., 2008). In *C*. *jejuni*, the frequency of recombination may be higher in genes which experience higher selection pressure, such as surface exposed structure encoding genes (Caro-Quintero et al., 2009), but recombination also occurs in conserved and essential housekeeping genes, which are expected to experience less selection pressure (Sheppard et al., 2008). The same dataset has been used to model the timescale of evolution for *C*. *jejuni, C*. *coli, C*. *fetus*, and several related *Campylobacter* species based on MLST (Wilson et al., 2009). This study indicated that speciation within the included *Campylobacter* species occurred on a timescale of thousands of years and it was speculated that the *C*. *jejuni*–*C*. *coli* split coincided with the Neolithic domestication of a wide variety of animal species around 10,000 years ago. This was in contrast to conventional estimates, which indicated a timescale of millions of years for *Campylobacter* evolution (Wilson et al., 2009).

## Evolution of *Campylobacter fetus*

Some studies have focused on the evolution of *C*. *fetus* (Tu et al., 2005; Kienesberger et al., 2014; Gilbert et al., 2016b; van der Graaf-van Bloois et al., 2016). This species consists of two genetically divergent and coherent lineages, each with a distinct host association, either mammal- or reptile-associated. While the subspecies *C*. *fetus* subsp. *fetus* is predominantly isolated from mammals, mostly ruminants, and *C*. *fetus* subsp. *venerealis* is restricted to cattle, *C*. *fetus* subsp. *testudinum* is predominantly isolated from reptiles. It was questioned whether this distinct host dichotomy was the result of divergence and subsequent co-evolution of mammal- and reptile-associated *C*. *fetus* with their respective mammalian and reptilian hosts since their last shared ancestor or whether this host-association was the result of a more recent host crossing event.

The large genetic diversity of reptile-associated *C*. *fetus* subsp. *testudinum* in reptiles indicates that the reptilian host is likely a more ancient reservoir for *C*. *fetus* than the mammalian host. The large genetic divergence between mammal and reptile-associated *C*. *fetus* suggests that this is not a recent host-crossing event and reptile-associated *C*. *fetus* subsp. *testudinum* is not the direct ancestor of mammal-associated *C*. *fetus* (Gilbert et al., 2016b). Furthermore, the isolation of *C*. *fetus* ST43 and ST69, another divergent *C*. *fetus* lineage which is more related to mammal- than to reptile-associated *C*. *fetus* from chelonians, indicates that *C*. *fetus* diversity is larger than foreseen (Wang et al., 2013; Gilbert et al., 2014, 2018). Based on 16S rRNA and whole genome sequence-based homology, these strains and mammal-associated *C*. *fetus* have a more recent common ancestor. Based on current knowledge, *C*. *fetus* diversity is larger in reptiles, which may be the primary *C*. *fetus* reservoir from which mammal-associated *C*. *fetus* originated. The finding of additional divergent *C*. *fetus* strains can provide further insights in *C*. *fetus* evolution, but also stresses that evolutionary insights can be largely influenced by screening efforts. Large-scale screening of wild mammals and reptiles is needed to fill in the gaps in *C*. *fetus* evolution.

While allopatric speciation is considered the most common form of speciation in organisms that tend not to disperse over large distances, this is often inferred to be a rare event in the evolution of bacteria (Papke and Ward, 2004). This is largely based on the assumptions that bacteria are small, have large population sizes, and have the ability to survive hostile environments in a resistant physiologically inactive stage during transport. However, while many of these assumptions apply to free-living bacteria, this only partly applies to *C*. *fetus* and other Epsilonproteobacteria adapted to vertebrate host with a low dispersal capacity. Furthermore, vertebrate-associated Epsilonproteobacteria do not form endospores and are highly sensitive to desiccation. Nevertheless, some Epsilonproteobacteria, like mammal-associated *C*. *fetus*, have shown to be genetically uniform across the globe (van Bergen et al., 2005). In this case, anthropogenic influences have likely blurred the original distribution patterns, as mammal-associated *C*. *fetus* has been transported together with their ruminant host across the globe as livestock (van der Graaf-van Bloois et al., 2016). One would expect some level of geographical isolation, reflected in the *C*. *fetus* genome. However, host association of *Campylobacter* genotypes has been shown to transcend geographic variation for *C. fetus* and *C. jejuni* (van Bergen et al., 2005; Sheppard et al., 2010; Griekspoor et al., 2013). This indicates that, at least for these species, host type is more important than geographic location in explaining genotypic variation. For example, on different continents similar MLST sequence types are observed for *C. fetus* and *C. jejuni* in cattle and poultry, respectively (van Bergen et al., 2005; Sheppard et al., 2010). Another interesting finding is that blackbirds from Europe and Australia (where they have been introduced) harbored similar sequence types (Griekspoor et al., 2013).

*C*. *fetus*, and *C*. *fetus* subsp. *venerealis* in particular, has a very high level of niche adaptation. This niche may be so narrow that many genetic adaptations will be detrimental and disadvantageous for niche persistence, confining the number of genetic variants. Nevertheless, it has to be noted that 16S rRNA and the MLST housekeeping genes are highly conserved and no or little genetic variants are observed in mammal-associated *C*. *fetus*. This indicates that mammal-associated *C*. *fetus* is coherent and expanded recently in the agricultural niche, allowing little time for diversification (Gilbert et al., 2016b; van der Graaf-van Bloois et al., 2016). Compared to other *C*. *fetus* variants, *C*. *fetus* subsp. *venerealis* displays the largest accessory genome due to the acquisition of non-homologous genetic material. This might be related to a less effective defense against foreign DNA, such as the absence of a complete CRISPR/Cas system (Gilbert et al., 2016b). Parts of the accessory genome are thought to be specific for *C*. *fetus* subsp. *venerealis* and might confer the cattle restricted niche specificity (Kienesberger et al., 2014). Gene acquisition by lateral gene transfer might have enabled *C*. *fetus* subsp. *venerealis* to exploit a slightly different niche. By adaptation to this niche, *C*. *fetus* subsp. *venerealis* could escape the force of cohesion with *C*. *fetus* subsp. *fetus* and start diverging by accumulation of point mutations. The force of cohesion is likely a combination of homologous recombination and purging of less adapted genetic variants. Indeed, this divergence has been observed in the core genomes of *C*. *fetus* subspecies *fetus* and *venerealis* (van der Graaf-van Bloois et al., 2014). The adaptation to different and often cryptic ecological niches has been demonstrated for *C*. *jejuni*, in which the two major generalist lineages did not show evidence of recombination with each other in nature, despite having a high degree of host niche overlap and recombining extensively with specialist lineages (Sheppard et al., 2014). However, transformation experiments showed that the generalist lineages readily recombine with one another *in vitro*. This suggested ecological rather than essential barriers to recombination, caused by a cryptic niche structure within the hosts. The large genetic distance observed between mammal- and reptile-associated *C*. *fetus* indicates prolonged divergence of both lineages (Gilbert et al., 2016b). The rarity of recent homologous recombination observed between mammal- and reptile-associated *C*. *fetus* supports effective isolation of both lineages and indicates that there are barriers to genetic exchange. Such barriers can be divided into three categories: mechanistic, ecological, and adaptive (Sheppard et al., 2008). As these *C*. *fetus* lineages inhabit either mammals or reptiles, an ecological barrier, resulting from the occupation of distinct niches, is likely most important. This is supported by the observation that *C*. *fetus* ST69, a lineage highly related to mammal-associated *C*. *fetus* subsp. *fetus*, yet isolated from chelonians, shows high recombination rates with reptile-associated *C*. *fetus* subsp. *testudinum* when occurring in a shared chelonian host (Gilbert et al., 2018). In contrast, gene flow between *C*. *fetus* and *C*. *iguaniorum*, which are both occurring in the same reptilian host, was rare, suggesting effective barriers to recombination despite having a similar niche (Gilbert et al., 2018). Depending on the frequency of homologous recombination, bacteria can range from either clonally to sexually reproducing organisms (Fraser et al., 2007). Within *C*. *fetus*, mutation through homologous recombination has been shown more important than mutation through point mutation in reptile-associated *C*. *fetus* subsp. *testudinum*, compared to mammal-associated *C*. *fetus* subspecies *fetus* and *venerealis* (Gilbert et al., 2016b). Also, recombination has likely disrupted the association between genome phylogeny and *sap* type in reptile- but not in mammal-associated *C*. *fetus*, as both *sap* types can be present in some clades in *C*. *fetus* subsp. *testudinum*, while only one *sap* type is present in each clade in *C*. *fetus* subspecies *fetus* and *venerealis*. As such, *C*. *fetus* subsp. *testudinum* can be considered to reproduce more sexually, whether *C*. *fetus* subspecies *fetus* and *venerealis* show more clonal reproduction. In stable and rather static environments a clonal reproduction mode can be favorable, whereas in unstable and dynamic environments a sexual reproduction mode can be more favorable for survival, providing the variation needed for selection to act upon (MacLean et al., 2013). For *C*. *fetus*, this can be explained from an ecological perspective, as mammal-associated *C*. *fetus* currently appears to be primarily restricted to a rather narrow host niche in an agricultural setting, whereas reptile-associated *C*. *fetus* shows a broad host niche amongst various reptile lineages, as diverse as squamates (lizards and snakes), chelonians, and crocodilians (Gilbert et al., 2014). The large genetic diversity observed in *C*. *fetus* subsp. *testudinum* supports an ancient introduction in the reptilian host, although intrinsic factors generating this genetic diversity cannot be excluded. Although both mammals and reptiles are likely occasionally exposed to both mammal- and reptile-associated *C*. *fetus*, and both lineages show the same growth temperature range, the distinct host dichotomy maintains, suggesting effective host adaptation of either lineage.

Unlike in most *Campylobacter* species, a proteinaceous S-layer is present in *C*. *fetus*, encoded by *sap* genes, which can be *sapA, sapB*, or occasionally *sapAB*. These *sap* variants correlate with the lipopolysaccharide composition, which can either be serotype A or B, with serotype AB being a minor variant of serotype B (Blaser et al., 2008). As most reptile-associated *C*. *fetus* were type A, it was suggested that this was the ancestral type, after which type B diverged in mammals (Tu et al., 2005; Kienesberger et al., 2014). However, the subsequent discovery of type B strains in reptile-associated *C*. *fetus* suggests that both types originated before reptile- and mammal-associated *C*. *fetus* diverged (Gilbert et al., 2016b). It remains to be shown how both types have remained conserved over time in both divergent reptile- and mammal-associated *C*. *fetus* lineages.

Although co-evolution of mammal and reptile-associated *C*. *fetus* with their respective mammalian and reptilian hosts since their last common ancestor has been suggested (Tu et al., 2001), this has been refuted by others based on evolutionary rate modeling (Wilson et al., 2009), and, based on current knowledge and species distribution, a more recent crossing event from a reptilian, or from another, unknown host, to a mammalian host seems more plausible. Indeed, considering the ancient split of mammals and reptiles, 320–315 million years ago (Mya), and the massive radiation and diversification of species afterward, effective co-evolution between *Campylobacter* and their hosts would be reflected in a higher diversity in *C*. *fetus*, much more genetic divergence between mammal- and reptile-associated *C*. *fetus*, and to another phylogenetic composition of the *Campylobacter* genus as a whole, especially considering the short generation time and high mutation rate normally encountered in bacteria. A stable intestinal environment could provide protection and promote genetic stability over time. However, physiology of the mammalian and reptilian intestinal tract has evolved in separate ways as well. This diversity of distinct intestinal niches is not reflected in the currently observed *Campylobacter* diversity.

There is little theoretical support for the timescale hypothesized by Tu et al. (2005). However, there are large differences in the evolutionary timescales, depending on the molecular clock model considered. Based on *Campylobacter* MLST data, the split between *C*. *fetus* and *C*. *jejuni* was calculated to have occurred 33.8 Kya, whereas this split occurred 51.4 Mya according to the most relaxed model (Wilson et al., 2009). Based on whole genome sequence data, it has been shown that diversification of mammal-associated *C*. *fetus* is a relatively recent event, as the split of the most basal clades was predicted to have occurred 10.5 Kya to more than 40 Kya (van der Graaf-van Bloois et al., 2016; Iraola et al., 2017). When compared to related vertebrate-associated Epsilonproteobacteria species, the model proposed by Wilson et al. (2009) might be too strict and the timescale proposed for *Campylobacter* evolution too narrow. For example, *H*. *pylori* has been co-evolving with humans and diversity reflects human ancestry and migration patterns out of Africa an estimated 50–70 Kya (Moodley et al., 2012). This indicates that *H*. *pylori* diversified extensively during this timescale, but diversity has not crossed the species boundaries. According to the molecular clock proposed by Wilson et al. (2009), *Campylobacter* evolved into at least 13 separate species within a similar timescale. Although *Campylobacter* and *Helicobacter* might show intrinsic differences which influence the speciation rate, such large differences are not to be expected.

In all likeliness, a reliable bacterial molecular clock for longer evolutionary timescales is not realistic without calibration based on ancestral reference points. Processes like lateral gene transfer can potentially change the evolutionary trajectory of all bacteria with only a single gene transfer event. Due to ancestral interspecies lateral gene transfer, the evolutionary trajectory of each gene can potentially be different from the evolutionary trajectory of the bacteria in which the gene resides at a particular time. In that way a molecular clock would rather predict the evolutionary timescale of a gene or genome than of the organism itself.

## Epilogue

Reptiles harbor an unexpectedly high Epsilonproteobacteria diversity and many of these taxa are uniquely associated with reptiles. Especially *Helicobacter* shows extensive host adaptation and diversification within the reptilian host. Despite the advances made, many questions remain largely unanswered, such as the Epsilonproteobacteria prevalence and transmission in wild reptiles, pathogenicity of Epsilonproteobacteria in reptiles, the factors determining host adaptation, and the timescale of Epsilonproteobacteria evolution. Future studies are needed to address these questions in further depth.

## Author Contributions

MG wrote the manuscript. BD, AZ, and JW reviewed the manuscript.

## Conflict of Interest Statement

The authors declare that the research was conducted in the absence of any commercial or financial relationships that could be construed as a potential conflict of interest.

## Footnotes

1 www.bacterio.net/index.html
